# System Development for Measuring Nursing Workload Based on the Real-Time Location Information

**DOI:** 10.1155/jonm/2188150

**Published:** 2025-09-17

**Authors:** Hyunggon Park, Younhee Kang

**Affiliations:** ^1^Department of Electronic and Electrical Engineering, College of Engineering, Ewha Womans University, Seoul, Republic of Korea; ^2^Graduate Program in System Health Science and Engineering, College of Nursing, Ewha Womans University, Seoul, Republic of Korea

**Keywords:** nurses, smartphone, workload

## Abstract

**Background:** There are no objective measurement methods that provide empirical data on nursing workload.

**Aim:** This study aimed to develop a system to measure nursing workload using beacons and a smartphone based on the real-time location information.

**Methods:** This was a study of the technological development of a system for measuring nursing workload using beacons and smartphones. The validation of the system developed was performed by assessing the recognition rate of beacons by multiple walk-through tests and determining the optimal number of beacons and smartphones for a higher recognition rate of beacons.

**Results:** The system consisted of four components: data-generation devices, nurse devices, servers, and databases. The location information generated from beacons is stored in nurses' smartphones. Then, these data were then transmitted from the smartphone to the server and stored in a server database. As a result of system validation, the optimal recognition rate of the beacon through the smartphone was determined as 97.5% and 96.2% with 12 beacons and 15 beacons, respectively, in one smartphone. The higher the number of beacons and smartphones employed in the system developed, the lower the recognition rates were.

**Conclusions and Implications for Nursing:** The system developed in this study for measuring nursing workload is highly accurate in providing nurses' real-time location information. Thus, this system could be utilized to measure the nursing workload objectively because it provides the real-time location information, moving path, and distance of nurses during work.

## 1. Introduction

Nurses are the largest group of healthcare professionals and account for more than 50% of the healthcare workforce [1]. The total global nursing workforce is 27.9 million, including 19.3 million (69%) professional nurses, 6.0 million (22%) associate professional nurses, and 2.6 million (9%) who are not classified [1]. Nurses play a pivotal role in achieving universal health coverage and the Sustainable Development Goals (SDGs) [1]. However, according to the world's nursing reports [1], the global distribution and size expansion of the nursing workforce are inequitable and inadequate to meet the rising demand. South Korea has experienced a chronic nursing shortage.

The overall turnover rate of Korean hospital nurses was 15.4%, and the mean number of years of service was only 5.4 years. Moreover, the turnover rate of newly graduated nurses within one year was reported to be 45.5% in 2019 [2]. The number of nurses per 1000 per capita in Korea was 3.6 nurses, which was about half of the mean number of Organization for Economic Co-operation and Development (OECD) member countries (6.5 nurses per 1000 capita), and only 51.9% of licensed nurses actively work in clinical areas [3]. Even with this number of nurses, the total number of hospital beds in Korea is more than three times the mean number of hospital beds in OECD member countries. Therefore, the number of patients assigned to a nurse in each shift is two to five times higher than that in other developed countries [3], and their workload is expected to be higher than that in any other country.

The Korean government increased the entrance quota of nursing schools from 12,573 in 2009 to 20,033 in 2019 to increase the number of newly graduated nurses yearly [4]. Despite this measure of increasing the number of new nurses, the problem of nursing workforce shortages has not been solved. The solution for the nursing workforce shortage should not be the production of new nurses but the prevention of current nurses' turnover. A high level of nurse turnover induces a nursing workforce shortage, which may not be able to provide patients with appropriate nursing care [5]. Consequently, high nurse turnover and low quality of nursing care may significantly threaten patient recovery and health. Working conditions have been investigated to prevent nurses' turnover and to retain them. The main causes of nurses' turnover are their working conditions: overworkload, low wages, role conflict, and uncomfortable institutional culture [6]. Among these factors, nurses' overwork was the most influential reason for turnover. Thus, quantifying nursing workload is one of the main antecedents in suggesting an appropriately balanced allotment of hospital nurses to relieve their overworkload. Unfortunately, quantification of nursing workload using scientific methods has not yet been achieved.

To estimate the actual workload of hospital nurses, a few measurement methods of nursing workload have been utilized, including the estimation of nursing hours based on nursing care needs by the Korean Patients Classification System (KPCS) for nurses [7, 8], direct observation of each nursing activity with a stopwatch [7, 9], and 24-h recall with the Nursing Activity Score (NAS) only for intensive care units [10–12]. The KPCS does not clearly acknowledge nursing hours; on the other hand, it only considers the number of nursing care sessions and patients' nursing needs. The NAS was developed to measure nursing workload only for intensive care unit settings, so that the nursing workload of other nursing settings could not be captured. Likewise, these methods of measurement have been limited in terms of observer bias, Hawthorne effect from direct observation, incomplete recall, and inability to collect information on the travel distance and time [7, 9, 11, 12]. From findings from the previous studies, the diverse factors were shown as influencing the nursing workloads, including patient acuity, staffing levels, administrative responsibilities, and use of technology [11, 12]. In order to draw a comprehensive and integrated knowledge of nursing workload, a universal and objective measure was required.

To suggest a proper nursing workforce arrangement, a new objective method for measuring nursing workload using fourth industrial revolution technology should be introduced for diversified nursing settings. The current technology status of beacons could locate nurses during work using real-time spatial information [13–18].

These data could be recorded per second throughout the working hours. The recorded data were then transferred to and stored on a data server. However, until now, there has been no trial to develop a system to locate nurses during work and to measure nursing workload using the real-time location information through this technology of beacons.

## 2. Purpose

From this perspective, this study aimed to develop a system to measure nursing workload using beacons and a smartphone based on the real-time location information. The system was developed under the research project “Surveillance and Measurement for Assessing Real Tasks in Nursing Using Real-Time Systems and Enabled Technologies (SMART-NURSE).” Specific aims of the study were to develop a system to measure nursing workload and to validate the system developed, where the real-time location information collected was reflected on the actual movement path.

The first part of this study, “background of system development to measure nursing workload” and “development of system,” was previously published in a preprint [19].

## 3. Methods

### 3.1. Development of a System

This was a study of the technological development of the system for measuring nursing workload using beacons and smartphones. Beacons were employed for location data, and the data were collected using a Bluetooth communication system between beacons and smartphones. Finally, data transmissions among devices and the data server were conducted through socket connections.

The beacon selected for this study was a new generation beacon, E7, standard iBeacon mounting Bluetooth 5.0 hardware platform made by Hyunseung Korea, South Korea. This beacon simultaneously transmits iBeacon, Eddystone (UI, URL, and TLM), and sensor data. The maximum transmission power (range) was 100 m. The shape and size of the beacon were round and 39 mm in diameter and 15.5 mm in height.

Two mobile applications (“beacon–iPhone” application and “iPhone–server” application) were developed to collect the location information from beacons and transmit it to the database on the server.

### 3.2. Validation of the System Developed

**Figure 1:**
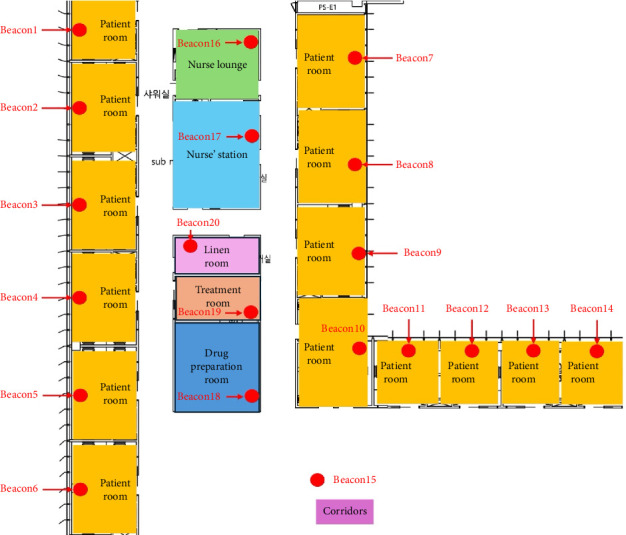
Layout of beacons' installation.

In order to evaluate the accuracy of the system developed, a medical unit of a general hospital was designated, and different numbers of beacons were installed in each patient's room, corridors, treatment room, drug preparation room, nurses' station, linen room, and nurse's lounge. Comparisons of the frequency of beacons' recognition in the smartphone with the number of beacons in real movement paths during multiple walk-through tests were conducted to assess the recognition rate of beacons. At first, a total of 20 beacons were installed (Figure 1), and then the number of beacons installed was adjusted in accordance with the beacons' recognition rate. In addition, the optimal number of smartphones was adjusted in order to increase the recognition rate of beacons.

## 4. Results

### 4.1. Overview of the System Developed

**Figure 2:**
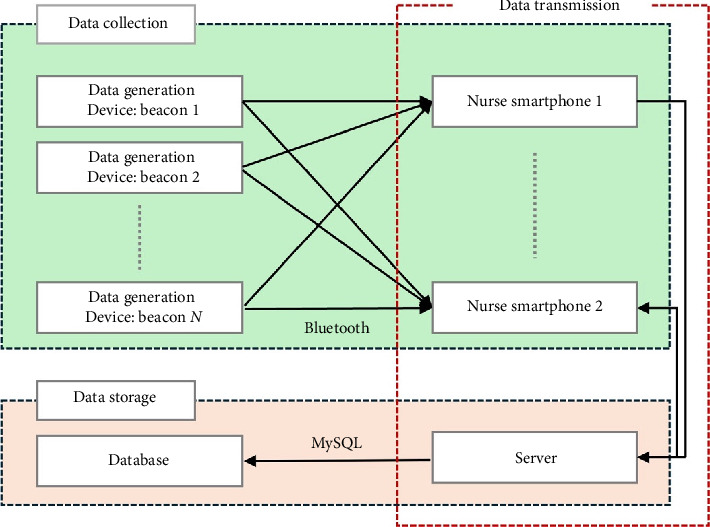
Overview of system implementation.

The overall system comprised four components: data-generation devices (beacons), nurse devices (smartphones), servers, and databases. The location data generated from beacons are stored in the nurses' smartphones. The data were then transmitted from the smartphone to the server and stored in a server database. An overview of the system implementation is shown in Figure 2.

The data required to determine the indoor location were generated from multiple beacons installed in several places (patient's room, corridors, treatment room, drug preparation room, nurses' station, linen room, and nurse lounge). The location data in the beacons were transmitted to individual smartphones. The data in the smartphone are again delivered to the server and stored in a database.

### 4.2. System Implementation

In this section, we describe how the proposed system is implemented, where the data collected from beacons are transmitted to a database on the server via nurses' smartphones.

#### 4.2.1. Location Data Collection

Location data are collected from multiple beacons if the nurses' smartphones are in proximity to the beacons. Since beacons are passive, the data acquisition process must be implemented as a “beacon–iPhone” application on smartphones. The detailed implementation steps are as follows:

  Step 1: The existing database is imported with the application executed on a smartphone. If there is no table for storing the delivered data, a new table can be created. In our implementation, the database table consists of seven columns: current time, device names for data collector identification, major and minor values for beacon identification, proximity, accuracy, and received signal strength indicator (RSSI) values.
  Step 2: Receive *N* nearest beacon signals with a prespecified UUID value through beacon scanning. In our implementation, we set *N* to 3, that is, three beacons.
  Step 3: Insert the collected data into the table in the database. Steps 2 and 3 are repeated until the application is closed.

#### 4.2.2. Location Data Transmission to the Database

For data transmission, we adopted socket communication to transmit the data to the server as it supports real-time, bidirectional, and event-based communications. In particular, the implementation requires bidirectional communication between the server and the smartphone while transmitting a large amount of data in real-time. The detailed implementation of the location data transmission steps is described as follows:

  Step 1: A socket connection is established for data transmission between the smartphone and the server. When using Port 3000 of the server in our implementation, any available port can be used.
  Step 2: With the established socket connection, the smartphone name (i.e., phone ID) is delivered to the server and used to query data from the database.
  Step 3: The server then sends information about the time of the most recent stored data to the database every 10 s. This information is stored as the variable timestamp.
  Step 4: The researcher reads the beacon data table, sends it to the server as a message event, and removes it from the table. The server then inserts the data received during the message event into the database. The location data include information about time, major, minor, proximity, accuracy, RSSI, and the nurse's device (smartphone). This step is continued until the last row is read and transmitted.
  Step 5: If termination is requested by pressing the button in the application, and the timestamp of the last time and message is identical, the socket connection is terminated.

### 4.3. System Execution

**Figure 3:**
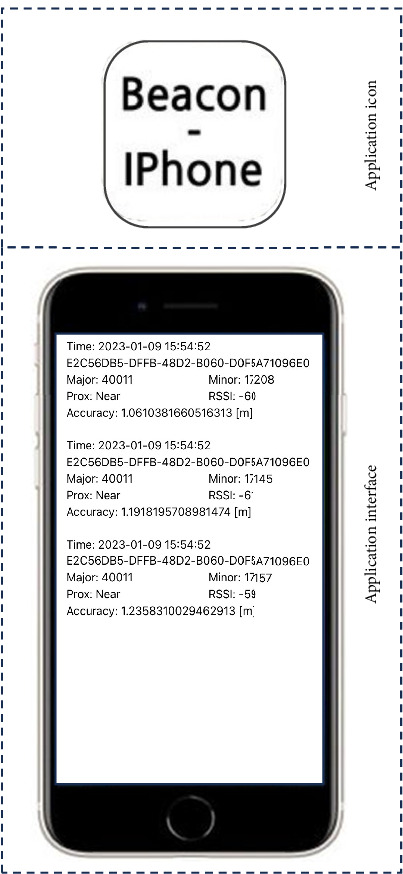
Beacon–iPhone application.

The system developed in this study was preliminarily installed and implemented. Beacons were located in each patient's room, corridor, treatment room, drug preparation room, nurses' station, linen room, and nurses' lounge in the medical unit (Figure 1). A total of 20 beacons were installed where the nurse might travel during work duty. An application “beacon–iPhone” was opened on a smartphone (Figure 3), which was administered to a nurse. The nurse usually worked as he/she did with the smartphone. During the nurses' working duties, real-time location information was collected using the smartphone.

**Figure 4:**
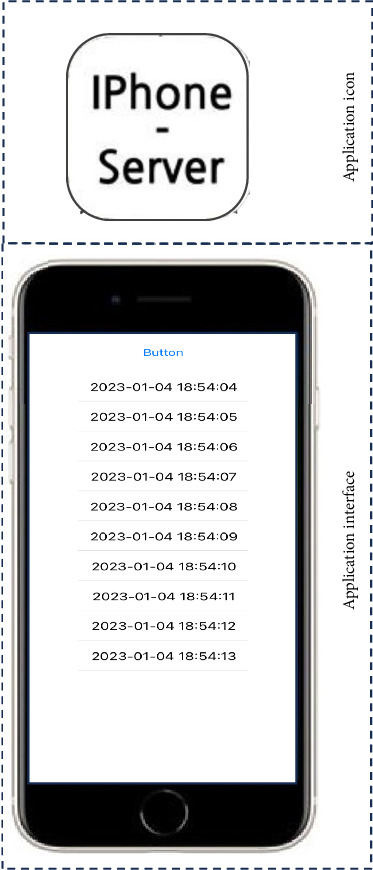
iPhone–server application.

Upon the completion of the nursing work duty, the “beacon–iPhone” application was closed, and the “iPhone–Server” application was opened (Figure 4); consequently, the data transmission to the server was conducted. All the data were stored in the study server and synchronized in real-time so that the duration that the nurse spent providing direct care, indirect care, and others during work, depending on the location where the nurse was placed or traveled, could be determined.

#### 4.3.1. Validation of the System Developed

Under the consideration of each space in a medical unit of a hospital and multiple times of pilot system execution, the transmission power of the beacon was adjusted to 50 m to prevent signal distortion. Because Bluetooth signals from the beacon are transmitted in radial form, the recognition distance is around 25 m with the transmission power of 50 m.

The results of multiple walk-through tests with the recognition rate of beacons are shown in Table 1. Under the condition of 20 beacons installed, the recognition rate of beacons was assessed with one, two, and three smartphones, respectively. With one smartphone, the recognition rate of beacons was 77.2%. With two smartphones simultaneously collecting location data, the recognition rate of beacons was 68.8% and 51.3% respectively, in each phone. The lower recognition rate of beacons was shown as 60.5%, 53.5%, and 48.0% with three smartphones collecting data at the same time. The higher the number of smartphones was applied, the lower the recognition rate of beacons was found. Thus, the numbers of beacons and smartphones were adjusted to 17 beacons and one or two smartphones. Under the 17 beacons installed, an 87.9% recognition rate was shown with one smartphone, and 73.5% and 70.6% recognition rates were shown with two smartphones simultaneously collecting location data. Since this level of recognition rate of beacons is not appropriate enough to adopt, the number of smartphones was set as one, and then the number of beacons was adjusted to 15, 12, and 10, respectively. 96.2%, 97.5%, and 98.8% recognition rates of beacons were shown in each of 15, 12, and 10 beacons installed.

## 5. Discussion

Throughout this study, a system measuring nurses' workload using beacons and a smartphone based on the real-time location information was developed and validated for its accuracy in measuring the real-time location information. The system developed was composed of data-generation devices (beacons), nurse devices (smartphones with two applications), servers, and databases. It collected, transmitted, and stored the real-time location information successfully.

Upon results of multiple walk-through tests with the recognition rate of beacons, the optimal number of beacons and smartphones simultaneously collecting real-time location information was determined. With the smaller number of beacons, the recognition rate of beacons was much higher in one smartphone rather than in two or three smartphones. However, the areas where a nurse is usually walking around during a work shift are generally defined, including patients' rooms, corridors, treatment room, drug preparation room, nurses' station, linen room, and nurse's lounge. In addition, the number of patients and patients' rooms to be cared for by a nurse are always determined as a certain number. Therefore, we concluded that 15 or less beacons with one smartphone were determined as the most appropriate number of them, although the best recognition rate of beacons with one smartphone was shown with the 10 beacons installed, which could not cover all the areas where a nurse walks around during work.

The consideration of the number of beacons and smartphones would be a pivotal decision under the complex and dynamic hospital settings with a high traffic of electronic signals and wifi interference. The higher recognition rate of beacons represents the higher accuracy of the system developed in measuring nursing workload under the real nursing care situations. Therefore, the findings of this study hold significant value as they provide an optimal system evaluation that genuinely reflects the realities of clinical nursing practice.

There have been no previous research studies that developed a system where the nurses' working movement path is tracked and evaluated for its accuracy in providing real-time location information. Therefore, this study is the first system development for the purpose of measuring nursing workload using beacons and smartphones. Through this system developed, the objective quantification of nursing workload by time and location during nursing work duties might be actualized. This system employs future-promising technology and will be able to produce differentiated data on nursing workload compared to that measured through direct observation or recall. This measurement system will resolve problems (e.g., observer bias, Hawthorne effect from direct observation, and incomplete recall) in existing methods of measuring nursing workload.

The data produced from the system developed in this study would be accumulated and stored in the server in seconds, and the nursing workload for 24 h could be measured repeatedly using real-time location information. The collected data are called Big Data and are used for further analyses, such as machine learning and AI algorithms.

Furthermore, based on accurate data on nursing workload by type of hospital, department, and work shift, a prediction model of nursing workload could be developed using Big Data and AI. Big Data collected with mobile devices can be analyzed automatically with AI or machine learning algorithms; moreover, diverse inferences might be possible. For example, the characteristics of each nursing unit were determined, and the nursing workload for each nursing unit could be identified. Based on these results, a more precise and reasonable nursing workforce arrangement could be suggested.

### 5.1. Limitations

In this study, a system was developed using mobile technology and beacons to comprehensively measure nursing workload. The applications developed were based on the platform of iOS system so that the interoperability with Android devices would be a limitation. To enhance usability, it is essential to develop applications that are compatible with both Android devices and the iOS platform, or to create a dedicated platform for Android devices. In addition, another limitation of the study is that the accuracy of the real-time location information collection system may vary depending on wifi interference and the layout of the units in the hospital. However, these limitations could potentially be addressed through pilot testing prior to data collection.

## 6. Conclusion

This study aimed to develop a system to measure nursing workload using wearable devices and real-time location information. The developed system comprises four components: data-generation devices (beacons), nurse devices (smartphones with two applications), servers, and databases. Beacons and two applications in smartphones were used to collect real-time location data. Data collection was performed using a Bluetooth communication system, and data transmission among the devices and data servers was conducted through socket connections.

As a result of validation of the developed system with multiple walk-through tests, the 15 or less beacons with one smartphone were determined with the recognition rate of 97.5–96.2% to measure nurses' movement path practically. Through this system, objective quantification of nursing workload might be possible. Furthermore, based on Big Data in seconds of quantified nursing workload by types of hospitals, departments, and work shifts, a prediction model of nursing workload can be developed. For example, it would be possible to derive patterns of nursing workload based on objectively measured data from the developed system, categorized by shift, department, and hospital level. Through machine learning applications focused on nursing workload patterns, it will be possible to develop predictive algorithms and artificial intelligence models tailored to nursing workloads across different shifts, departments, and hospital tiers. This advancement is anticipated to provide objective evidence to support effective nursing staff allocation.

### 6.1. Implications for Nursing

One of the main roles of nurse managers is to appropriately arrange the nursing workforce. This manpower arrangement in a healthcare environment is the most important factor, not only in providing optimal healthcare services but also in retaining experienced manpower to guarantee high-quality healthcare services. The exact nursing workload should be obtained to estimate the appropriate nursing staffing level. The system measuring nursing workload using real-time location information could be one solution to estimate the nursing workload as the fundamental stage in planning the proper nursing staffing level. Furthermore, the precise prediction of nursing staffing levels according to the types of hospitals and departments could be produced based on inferences on the workforce from AI algorithms with Big Data on nursing workload. Thus, nursing managers can estimate and apply the best nursing staffing levels and recommend them to health policymakers.

## Figures and Tables

**Table 1:** Recognition rate of beacons by the number of beacons and smartphones.

Number of beacons	Number of smartphones		
	1	2	3
	% (recognition frequency/actual frequency of movement path)		
20	77.2% (556/720)_smartphone 1	68.8% (220/320)_smartphone 1 51.3% (164/320)_smartphone 2	60.5% (121/200)_smartphone 1 53.5% (107/200)_smartphone 2 48.0% (96/200)_smartphone 3
17	87.9% (478/544)_smartphone 1	73.5% (225/306)_smartphone 1 70.6% (216/306)_smartphone 2	—
15	96.2% (202/210)_smartphone 1	—	—
12	97.5% (234/240)_smartphone 1	—	—
10	98.8% (247/250)_smartphone 1	—	—

## Data Availability

The data that support the findings of this study are available on request from the corresponding author. The data are not publicly available due to privacy or ethical restrictions.
